# Pattern of Antimicrobial Susceptibility and Antimicrobial Treatment of Neonates Admitted with Suspected Sepsis in a Teaching Hospital in Ghana, 2021

**DOI:** 10.3390/ijerph191912968

**Published:** 2022-10-10

**Authors:** Kwaku Anim Omenako, Anthony Enimil, Afia Frimpomaa Asare Marfo, Collins Timire, Palanivel Chinnakali, Ama Pokuaa Fenny, Kathiresan Jeyashree, Kwame Ohene Buabeng

**Affiliations:** 1 Eastern Regional Hospital, Ghana Health Service, Koforidua P.O. Box KF 201, Ghana; 2 Department of Pharmacy Practice, Kwame Nkrumah University of Science and Technology (KNUST), Kumasi 00233, Ghana; 3 Komfo Anokye Teaching Hospital (KATH), Kumasi P.O. Box KS 1934, Ghana; 4 School of Medicine and Dentistry (SMD), Kwame Nkrumah University of Science and Technology (KNUST), Kumasi 00233, Ghana; 5 Centre for Operational Research, International Union Against Tuberculosis and Lung Disease, 68 Boulevard Saint Michel, 75006 Paris, France; 6 Department of Preventive and Social Medicine, Jawaharlal Institute of Postgraduate Medical Education & Research (JIPMER), Puducherry 605006, India; 7 Institute of Statistical, Social and Economic Research (ISSER), University of Ghana, Legon, Accra P.O. Box LG 25, Ghana; 8 Department of Epidemiology and Biostatistics, National Institute of Epidemiology, Chennai 600077, India

**Keywords:** antimicrobial resistance, neonatal sepsis, prescription, compliance, empirical antimicrobial treatment, standard treatment guidelines, SORT IT, operational research

## Abstract

Neonatal sepsis is a life-threatening emergency, and empirical antimicrobial prescription is common. In this cross-sectional study of neonates admitted with suspected sepsis in a teaching hospital in Ghana from January–December 2021, we described antimicrobial prescription patterns, compliance with national standard treatment guidelines (STG), blood culture testing, antimicrobial resistance patterns and treatment outcomes. Of the 549 neonates admitted with suspected sepsis, 283 (52%) were males. Overall, 529 (96%) received empirical antimicrobials. Most neonates (*n* = 407, 76.9%) were treated empirically with cefuroxime + gentamicin, while cefotaxime was started as a modified treatment in the majority of neonates (46/68, 67.6%). Only one prescription complied with national STGs. Samples of 257 (47%) neonates underwent blood culture testing, of which 70 (27%) were positive. Isolates were predominantly Gram-positive bacteria, with coagulase-negative Staphylococcus and Staphylococcus aureus accounting for 79% of the isolates. Isolates showed high resistance to most penicillins, while resistance to aminoglycosides and quinolones was relatively low. The majority of neonates (*n* = 497, 90.5%) were discharged after successfully completing treatment, while 50 (9%) neonates died during treatment. Strengthening of antimicrobial stewardship programmes, periodic review of STGs and increased uptake of culture and sensitivity testing are needed to improve management of sepsis.

## 1. Introduction

Neonatal sepsis is a life-threatening organ dysfunction caused by a dysregulated host response to infection in the first 28 days of life [1]. It is a major global health threat with high incidence and mortality rates in developing countries [2]. An estimated three million neonatal cases of sepsis occur annually worldwide [3]. Most of the deaths were reported from Sub-Saharan Africa and South Asia [4].

Early administration of appropriate antimicrobials is one of the most effective interventions to reduce mortality in neonatal sepsis. The imperative to provide antimicrobials as early as possible, however, must be balanced against the potential harms associated with administering unnecessary antimicrobials to patients without infection [1]. In many developing countries, broad-spectrum antimicrobials are often used empirically to treat neonatal sepsis as a result of poor access to diagnostic microbiology services. This results in the excessive empirical prescription of broad-spectrum antibiotics in many developing countries [5,6].

Against this background, there are increasing concerns regarding utility of empirical antimicrobials against increasing antimicrobial resistance. Antimicrobial resistance has worsened in the last decade, rendering most antibiotics obsolete [7,8]. Resistance to “reserve” antibiotics such as aztreonam, linezolid and minocycline has increased, with 50–70% of the common Gram-negative isolates now multidrug resistant [4].

Although the understanding of sepsis epidemiology has improved globally, there are still major knowledge gaps in antimicrobial prescription and susceptibility patterns in developing countries such as Ghana. A study conducted in 2016 on neonatal sepsis from Ho Municipal Hospital in Ghana reported the proportion of culture-proven neonatal sepsis to be 17.3%, and all the isolates showed resistance to ampicillin, one of the first-line agents [9]. Furthermore, low antibiotic susceptibility coverage for organisms causing bloodstream infections was reported in two teaching hospitals in Ghana when the national and WHO-recommended empirical antimicrobials were assessed [10,11]. Hence, there is a need for periodic review of first-line medicines prescribed for empirical treatment of neonatal sepsis in Ghana.

The Komfo Anokye Teaching Hospital (KATH), located in Kumasi, Ghana, is a tertiary care teaching hospital. In this study, in neonates admitted with suspected sepsis at KATH, Ghana, from January–December 2021, we described (a) antimicrobial prescription patterns and compliance with recommendations of the national standard treatment guidelines (STG) for the management of sepsis, (b) blood culture positivity rate and antimicrobial sensitivity pattern of bacterial isolates and (c) treatment outcomes at the time of hospital discharge.

## 2. Materials and Methods

### 2.1. Study Design

This was a cross sectional study involving a review of medical records of neonates (<28 days of age) admitted with suspected sepsis.

### 2.2. Study Setting

#### 2.2.1. General Setting

Ghana, a country with an estimated population of 30.8 million [12], is located in the West African region and is bounded on the north by Burkina Faso, on the east by Togo, on the south by the Atlantic Ocean and on the west by Côte d’Ivoire. An estimated 12.2 percent of Ghana’s total population are aged 0–4 years [12]. In 2018, sepsis and other infectious conditions of newborns accounted for 5.3% (175/3300) of all deaths in children under 5 years [13].

#### 2.2.2. Specific Setting

The Komfo Anokye Teaching Hospital (KATH) is located in Kumasi, the capital city of Ashanti, one of the sixteen administrative regions in Ghana. Kumasi, which is the country’s second-largest city, is predominantly urban. KATH is a tertiary referral healthcare centre that provides a wide range of specialist services to the middle belt of the country. The Child Health Directorate of the hospital offers specialist out-patient, emergency and in-patient services to children. The directorate has four in-patient wards. In addition, there is a paediatric emergency unit (PEU) and a Paediatric Intensive Care Unit (PICU). On average, about 7500 children are admitted in a year [14].

The majority of patients are subscribers of the National Health Insurance Scheme (NHIS), which pays for the cost of healthcare. A few of the services, such as dialysis for chronic kidney failure, angiography, echocardiography and organ transplantation, as well as medicines that are not on the NHIS Medicines List (such as amikacin, ceftazidime and vancomycin), are paid for by patients.

### 2.3. Management of Sepsis in the Study Setting

Ghana has a national STG document (seventh edition, 2017) that guides healthcare providers in deciding on appropriate treatments for common health problems. This has been adapted for use by KATH. An institutional antibiotic policy document is also available at the child health directorate, KATH, for guiding antibiotic prescriptions.

Generally, diagnosis of neonatal sepsis is based on clinical judgement and the systemic inflammatory response syndrome (SIRS). SIRS is defined objectively if any two of the four criteria listed here are present: (a) body temperature over 38 or under 36 degrees Celsius; (b) heart rate less than 100 beats/minute; (c) respiratory rate greater than 60 cycles per minute or partial pressure of carbon dioxide less than 32 mmHg; and (d) leucocyte count greater than 12,000/microliters or less than 4000/microliters or there are over 10% immature forms or bands [15,16]. Neonatal sepsis is classified by time of occurrence of infection as early onset sepsis (EOS) in the first 72 h of life and thereafter as late onset sepsis (LOS) [17]. Those who meet the criteria for sepsis are offered empirical treatment in accordance with the STGs for 5–7 days with ampicillin plus gentamicin or ampicillin plus cefotaxime (neonatal sepsis other than cord sepsis), cloxacillin plus gentamicin (neonatal cord sepsis) and ampicillin plus gentamicin plus metronidazole (neonatal bowel related sepsis) [16]. The facility-based antimicrobial protocol outlines penicillin/cefuroxime + gentamicin (first line), cefotaxime or ceftriaxone or piperacillin/tazobactam + amikacin (second line) and ceftriaxone + sulbactam (third line) as treatment recommendations for neonatal sepsis. The facility-based guidelines permit clinical judgement considerations for the use of penicillin (such as benzylpenicillin and ampicillin) in place of cefuroxime. Benzylpenicillin is preferred when intracranial involvement with *Streptococcus pneumoniae* infection is suspected, whereas ampicillin is used when *Listeria monocytogenes* infection is suspected. 

Blood samples are collected before starting empirical therapy using aseptic techniques by phlebotomists or doctors and sent to the laboratory for microbiological analysis. Depending on the suspected aetiology, other biological samples such as cerebrospinal fluid (CSF), urine and pleural fluid are also sent for culture. All isolated pathogens are tested for antimicrobial susceptibility by the Kirby–Bauer disc diffusion technique in accordance with the Clinical and Laboratory Standards Institute’s guidelines [18]. Results are expressed as susceptible, intermediate or resistant. Culture results are usually available within 5–7 days. These results are used to review patients and offer guided antimicrobial therapy. Demographic characteristics, antimicrobial prescriptions, type of biological samples, results of culture and sensitivity and hospital exit outcomes (discharged satisfactorily, absconded, discharged against medical advice, referred to other facilities, death) are routinely entered into the electronic health records (EHRs).

### 2.4. Study Population

All neonates (≤28 days) admitted with suspected sepsis at the Child Health Directorate of KATH from 1st January 2021 to 31st December 2021 were included in the study.

### 2.5. Data Variables, Data Sources and Data Collection

Data variables included patient registration number, date of birth (age), weight, sex, presenting clinical signs and symptoms, results of blood culture and sensitivity, antimicrobials used and their dosages and treatment outcomes. Data were extracted from the EHRs and exported to MS Excel 2016 (version 16.0).

Compliance with STGs was defined as the prescription of antimicrobials as recommended for the management of sepsis in the appropriate dosage regimen (dose, frequency, duration). If one of the above conditions was not met, the prescription was classified as non-compliant to STGs.

### 2.6. Data Analysis

Data were imported to EpiData Analysis software (version 2.2.2.186, EpiData Association, Odense, Denmark) for data cleaning and analysis. Categorical variables (e.g., blood culture results, antimicrobial susceptibility, antimicrobial prescription patterns and hospital exit outcomes) were presented as numbers and proportions. Continuous data were presented as means and standard deviations for normally distributed data and as medians and interquartile ranges for skewed data.

## 3. Results

### 3.1. Socio-Demographics and Clinical Characteristics of Neonates

The socio-demographic and clinical characteristics of neonates (<28 days) admitted for suspected sepsis are described in Table 1 and Table 2.

There was a total of 549 neonates with suspected sepsis, of whom 283 (52%) were males, and the majority (*n* = 385, 70%) were from urban settlements. The median (IQR) age of neonates was 1 (1–2) days. The majority of the neonatal sepsis cases (*n* = 463, 84%) were classified as EOS. The most common clinical manifestations at the time of admission were respiratory distress (250/514; 49%), fever (158/530; 30%), tachycardia (119/529; 22%) and low leucocyte count (119/423; 28%). Lethargy (49/532; 9%), seizures (39/536; 7%) and pale skin (29/535; 5%) were observed in some neonates.

### 3.2. Antimicrobial Use

#### 3.2.1. Empirical Antimicrobial Use

Gentamicin (*n* = 470, 88.8%), cefuroxime (*n* = 421, 79.6%), benzylpenicillin (*n* = 53, 10%) and cefotaxime (*n* = 52, 9.8%) were the most commonly prescribed empirical antimicrobials. Most of the neonates (*n* = 407, 76.9%) were treated with cefuroxime + gentamicin. Fifty-three (10%) neonates were given benzylpenicillin + gentamicin as empirical treatment, while forty-two (7.9%) neonates were given cefotaxime monotherapy (Table 3). A similar pattern of antimicrobial use was observed among the neonates that died; cefuroxime + gentamicin (22/50), benzylpenicillin + gentamicin (11/50) and cefotaxime (9/50).

The median (IQR) number of antimicrobials prescribed per patient was two (2–2). All medications prescribed to the neonates were administered intravenously. Prescribing by generic name was observed in all prescriptions. Only one patient’s prescription complied with the national STGs. Ninety-five percent (*n* = 502) of empirical treatment complied with institutional treatment guidelines (Table 4).

#### 3.2.2. Modification in Antimicrobial Use

Among the total of 549 neonates, antimicrobial therapy was modified in 68 (12.4%). Out of 68 neonates in whom therapy was modified, cefuroxime + gentamicin (*n* = 40, 58.8%), benzylpenicillin + gentamicin (*n* = 14, 20.9%) and cefotaxime (*n* = 9, 13.2%) accounted for the majority of the empirical prescriptions.

With regard to modifications in antimicrobial use, cefotaxime was started as a modified treatment in 46 (67.6%) cases. Ciprofloxacin was started as modified treatment in 13 (19.1%) cases, while the combination of ciprofloxacin + metronidazole was started as a modified treatment in three (4.4%) cases. Further multiple modifications were observed in a number of cases after the initial modifications (Table 5).

### 3.3. Blood Culture Testing

Overall, blood samples of 257 (47%) neonates were subjected to blood culture testing, of which 70 (27%) blood specimens were culture positive. Among them, *coagulase-negative Staphylococcus* (*n* = 34, 57%) and *Staphylococcus aureus* (*n* = 22, 37%) were the dominant Gram-positive species. The common Gram-negative species isolated were *Coliforms* (*n* = 4, 5.7%) and *Pseudomonas* (*n* = 3, 4.3%) (Table 6).

### 3.4. Resistance of Bacterial Isolates against Antimicrobials

Among the most common Gram-positive bacterial isolates (i.e., *coagulase-negative Staphylococcus* and *Staphylococcus aureus*) identified in this study, the highest level of resistance was observed against all tested penicillins and tetracycline. The resistance patterns of Gram-positive bacteria isolates are presented in Table 7.

*Klebsiella* (*n* = 2, 18%) isolate was resistant to ampicillin (*n* = 1, 100%) and all cephalosporins (*n* = 1, 100%) tested. *E. coli* (*n* = 1, 9%) isolates were sensitive to amikacin (*n* = 0, 0%), ciprofloxacin (*n* = 0, 0%) and levofloxacin (*n* = 0, 0%). *Coliform* (*n* = 4, 36%) isolates showed low levels of resistance against amikacin (*n* = 1, 33%), ceftriaxone (*n* = 1, 33%), cefotaxime (*n* = 1, 25%) and ciprofloxacin (*n* = 1, 25%). *Pseudomonas* (*n* = 4, 36%) isolates showed relatively low resistance to levofloxacin (*n* = 0, 0%), ciprofloxacin (*n* = 0, 0%) and amikacin (*n* = 1, 33%). The resistance patterns of Gram-negative bacteria isolates are presented in Table 8.

Out of the total number of 70 positive cultures, gentamicin was tested in 59 isolates, of which 28 (47.57%) were resistant, while amikacin was tested in 56 isolates, of which six (10.7%) were resistant. Similarly, levofloxacin was tested in 22 isolates, of which four (18.1%) were resistant, while ciprofloxacin was tested in 58 isolates, of which 14 (24.1%) were resistant. Cefuroxime, cefotaxime, ceftriaxone, ampicillin and cloxacillin showed higher levels of resistance against the bacterial isolates (Table 9).

### 3.5. Hospital Exit Outcomes

Out of 549 neonates admitted with suspected sepsis, the majority (*n* = 497, 90.5%) were discharged satisfactorily after successfully completing treatment. Nine percent (*n* = 50) died, and two (0.4%) were referred to other health facilities.

## 4. Discussion

The study showed a high use of unindicated antimicrobials and a relatively high resistance pattern to commonly used antimicrobials that were recommended by the national STGs for management of neonatal sepsis. A low percentage of blood culture and sensitivity testing and a low culture positivity rate were observed. The majority of the neonates were discharged after successfully completing treatment. These findings are discussed in detail in the following paragraphs.

Firstly, antimicrobial use is extremely common, even in the absence of significant symptoms and biochemical indicators of infection, as evidenced in our study. This finding is consistent with a similar study conducted in a tertiary hospital in Ghana where, of the 443 neonates who were prescribed antimicrobials, 53% were prescribed gentamicin [19]. With respect to the choice of antimicrobials, our analysis revealed that there was very low (<1%) compliance with national STGs. The dosages, however, were generally in compliance with standards, including the British National Formulary (BNF). Non-compliance with the recommendations of the national STGs appears justified, as the isolated pathogens showed a high level of resistance to most of the recommended penicillins. This questions the indication of ampicillin as a mainstay of neonatal sepsis treatment combinations in national STGs and WHO guidelines. The good susceptibility of isolates to gentamicin makes an aminoglycoside-based regimen a preferred antimicrobial option to treat neonatal sepsis. It is worth pointing out that an appreciable level of compliance (>90%) with the institutional treatment guidelines was observed in this study. This is in line with WHO recommendations that facility-based treatment guidelines for common infections based on local susceptibility data should be followed with continuous surveillance [20].

Secondly, the observation that less than fifty percent of the neonates with suspected sepsis had blood culture and sensitivity testing could be attributed to financial constraints, as this investigation was not covered under the NHIS. There were also reports that in many instances, the decision to carry out the test was made but not implemented, owing to logistical constraints at the facility’s medical laboratory. The importance of this test cannot be overemphasized. Conventional culture techniques remain the “gold standard” to confirm the diagnosis of neonatal sepsis [21]. Results of blood cultures play a critical role in informing regional empirical therapy guidelines and the tracking of antimicrobial susceptibility trends and patterns [22]. In an interventional study involving patients with bloodstream infections, integration of rapid diagnostics with antimicrobial stewardship resulted in decreased length of hospital length of stay, reduced total hospital costs and reduced mortality [23].

The finding of a relatively low culture positivity rate (27%) is similar to other studies in Ghana which reported a culture positivity of 22% and 26% [11,24]. This means that a considerable proportion of neonates prescribed antimicrobials did not have a bacterial infection, or they might have received antimicrobials prior to blood sampling. This was also the inference drawn by Fuch et al. (2018) and Schlapbach et al. (2022) in previous studies [5,25]. The high incidence of neonatal mortality can be linked to culture-proven EOS. Thus, a relatively low culture positivity rate explains why the overall treatment outcomes of neonatal sepsis cases were satisfactory.

Furthermore, the predominance of Gram-positive bacterial isolates identified is consistent with the observation by Sands et al. (2022) that they represent the most frequently reported cause of bacterial neonatal sepsis in low- and lower-middle-income countries [17,18]. Similarly, in a previous study in Ghana, CoNS were the most frequently isolated pathogens in early and late-onset infections [11]. Although CoNS are pathogenic, they are the most common human skin commensals that have been associated with contamination of blood cultures from poor sampling techniques. At the study site, CoNS are not routinely ruled out as contaminants. It is regarded as pathogenic and thus treated. In addition, biofilm formation is a key mechanism of their pathogenesis and is associated with elevated antimicrobial resistance. Standard antimicrobial susceptibility testing may not accurately predict the efficacy of an agent against biofilm-associated organisms. Further research on the role of biofilms in infection should enhance clinical decision making [26]. Thus, interpreting the clinical significance of these isolates can be challenging for physicians [27]. In contrast, Gram-negative bacteria were the predominant organisms isolated in similar studies elsewhere in South Asia [4,28]. Furthermore, Gram-negative infections have been associated with significantly greater morbidity and mortality in neonates and widespread AMR [4].

Thirdly, with regard to hospital exit outcomes, a greater proportion of neonates were discharged satisfactorily after successful completion of treatment. This was similar to an Ethiopian study where more than 80% of neonates were discharged after successfully completing treatment [29]. The relatively lower proportion of neonates who died was in contrast to higher proportions of 21% (Pakistan), 22% (Egypt) and 19% (Iran) reported in other studies [30,31,32]. The greater proportion of neonates discharged satisfactorily correlates with a relatively low culture positivity rate that suggests in turn that most neonates did not have an infection.

There are a number of strengths to this study. All neonates admitted with suspected sepsis were analysed, thereby eliminating potential bias resulting from sampling. The study adhered to the Strengthening the Reporting of Observational Studies in Epidemiology (STROBE) guidelines for reporting observational studies [33]. Moreover, this assessment involved routine operational data, and the findings are thus likely to reflect clinical reality of the study site. STGs and BNF were used to assess compliance with dosage recommendations. However, our study also has some limitations. Instances where there were insufficient data as well as missing data could be sources of bias. However, as such cases were limited in number, this is unlikely to have influenced the results. The small number of pathogens isolated in some instances did not permit a fair conclusion about the patterns of antimicrobial resistance found. Because the types of pathogens vary across localities, these findings should be generalized with care. Future multicentre studies will complement and validate our findings. There are important implications and recommendations from this study.

First, we recommend the strengthening of antimicrobial stewardship programmes in the facility. An antimicrobial stewardship aims to, among other things, optimise the treatment of infections, reduce adverse events due to antibiotics and reduce AMR [34]. We recommend a regular audit with feedback to prescribers on their prescription practices. An audit with feedback provides an opportunity for clinical staff to discuss their own prescribing practices, to identify priority areas for change and to set specific goals for themselves at the facility, department and/or ward level [20].

Second, we also recommend standardisation of the culture and testing procedure including use of a consistent set of locally available panels of antimicrobials so that recommendations of test results can be implemented clinically. Logistical constraints should also be addressed. Furthermore, addition of culture and sensitivity testing to the list of services covered by NHIS is recommended. Surveillance mechanisms, such as culture and sensitivity testing, are some of the important WHO recommendations to track antimicrobial resistance among agents causing infectious diseases [35].

Third, in order to ensure compliance with guidelines, we recommend regular review, on the basis of new evidence, of subsequent editions of the national and institutional STGs. There should be continuous education of practitioners on guidelines. As a teaching hospital, deviations from the treatment guidelines should be well explained in order to inculcate a good prescribing culture in trainees. Availability of electronic versions of treatment guidelines on mobile devices and laptops used by clinicians has proven to be an important intervention that can be emulated to ensure compliance.

Fourth, the fact that the majority of neonatal sepsis cases were EOS suggests vertical transmission. This calls for stringent infection prevention and control (IPC) practices among mothers and healthcare providers to prevent and control healthcare-associated infections (HAI). There is also the need to address both maternal and neonatal risk factors. In our study, notable among these risk factors were premature rupture of membranes (PROM), low birth weight (<2.5 kg) and exposure to potentially septic environments (deliveries outside health facilities). Poor waste management practices, especially at the community level, should also be addressed by the combined effort of all relevant stakeholders to tackle sources of infection.

Fifth, as efforts are being made to have EHRs replace manual patient folders nationwide, mechanisms to reduce missing data ought to be instituted: users should be trained to use the software effectively. An evaluation of the software’s architecture with a view to accommodating the various needs of the health sector is also recommended. This will enhance research activities, the implementation of which is one of the core mandates of teaching hospitals.

## 5. Conclusions

In this cross-sectional study, in neonates admitted with suspected sepsis in a teaching hospital in Ghana between January and December 2021, we found a high prevalence of antimicrobial use which was not compliant with national STGs. Less than half of neonates had culture and sensitivity testing performed, and this was associated with a relatively low culture positivity rate. Bacterial isolates were predominantly Gram-positive and showed high resistance to penicillins and most cephalosporins but moderate resistance to gentamicin, which is commonly used in sepsis antimicrobial therapy. The majority of the neonates had good treatment outcomes. We recommend the strengthening of antimicrobial stewardship programmes, regular review on the basis of new evidence of subsequent editions of the national STGs and inclusion of culture and sensitivity testing under the NHIS.

## Figures and Tables

**Table 1:** Socio-demographic characteristics of neonates admitted with suspected sepsis at Komfo Anokye Teaching Hospital, Ghana, between January 2021 and December 2021.

Variable	Characteristic	Number	Percentage
**Total**		549	(100)
Age groups (days)	0–3	463	(84.3)
	4–6	23	(4.2)
	7–13	28	(5.3)
	14–20	17	(3.1)
	21–28	17	(3.1)
	Median (IQR)	1	(1–2) days
Sex	Male	283	(51.5)
	Female	265	(48.3)
	Not recorded	1	(0.2)
NHIS subscriber	No	113	(20.6)
	Yes	433	(78.9)
	Not recorded	3	(0.5)
NHIS status (*n* = 436)	Active subscriber	184	(42.2)
	Non-active subscriber	249	(57.1)
	Not recorded	3	(0.7)
Residence	Urban	385	(70.1)
	Rural	138	(25.1)
	Not recorded	26	(4.7)
Length of hospital stay	≤7 days	449	(81.8)
	8–14 days	68	(12.4)
	>14 days	32	(5.8)
	Median (IQR)	4	(3–6) days

NHIS = National Health Insurance Scheme.

**Table 2:** Clinical characteristics of neonates admitted with suspected sepsis at Komfo Anokye Teaching Hospital, Ghana, between January 2021 and December 2021.

	Characteristic	Number	Percentage
**Total**		549	(100)
**Signs and Symptoms at admission**	Lethargy ^†^	49	(9.2)
	Seizures ^†^	39	(7.3)
	Pale skin ^†^	29	(5.4)
	Persistent diarrhoea/vomiting ^†^	13	(2.4)
	Reduced urine output ^†^	4	(0.8)
	Unconsciousness ^†^	3	(0.6)
	Body temperature *		
	Fever (>37.5 °C)	158	(29.8)
	Normal	305	(57.5)
	Hypothermia (<36.5 °C)	67	(12.6)
	Heart rate *		
	Tachycardia (>160 beats/minute)	119	(22.5)
	Normal	407	(76.9)
	Bradycardia (<100 beats/minute)	3	(0.6)
	Respiratory rate *		
	Normal (15–60 breaths/minute)	264	(51.4)
	Respiratory distress (>60 breaths/minute)	250	(48.6)
	Leucocyte count (10^9^ cells/L) *		
	Low (<10)	119	(28.1)
	Normal (10–20)	256	(60.5)
	High (>20)	48	(11.3)

^†^ There were multiple responses; * number of neonates with missing data for the following variables: lethargy = 17; seizure = 13; pale skin = 14; persistent diarrhoea/vomiting = 16; reduced urine output = 19; unconsciousness = 13; body temperature = 19; heart rate = 20; respiratory rate = 35; leucocyte count = 126.

**Table 3:** Distribution of empirical antimicrobials prescribed for neonates admitted for suspected sepsis at Komfo Anokye Teaching Hospital, Ghana, between January 2021 and December 2021.

Antimicrobial	Total *n* (%)
Gentamicin	470 (88.8)
Cefuroxime	421 (79.6)
Benzylpenicillin	53 (10.0)
Cefotaxime	52 (9.8)
Metronidazole	17 (3.2)
Ciprofloxacin	7 (1.3)
Ampicillin	1 (0.2)
Clindamycin	1 (0.2)
Combinations/Treatment regimens	*n* (%)
Cefuroxime + gentamicin	407 (76.9)
Benzylpenicillin + gentamicin	53 (10)
Cefotaxime	42 (7.9)
Cefuroxime + gentamicin + metronidazole	7 (1.3)
Cefuroxime + metronidazole	6 (1.1)
Cefotaxime + ciprofloxacin	3 (0.6)
Cefotaxime + gentamicin	3 (0.6)
Cefotaxime + metronidazole	2 (0.4)
Ciprofloxacin	2 (0.4)
Cefuroxime + metronidazole + ciprofloxacin	1 (0.2)
Cefotaxime + clindamycin	1 (0.2)
Ciprofloxacin + metronidazole	1 (0.2)
Ampicillin + cefotaxime	1 (0.2)

Denominator for all percentages = 529 neonates who received empirical antimicrobial treatment.

**Table 4:** Antimicrobial prescription practices and patterns among neonates admitted with suspected sepsis at Komfo Anokye Teaching Hospital, Ghana, between January 2021 and December 2021.

Parameter	*n*	(%)
Number (%) of patients prescribed no antibiotic	20	(3.6)
Number (%) of patients prescribed one antibiotic	44	(8.0)
Number (%) of patients prescribed two antibiotics	477	(86.9)
Number (%) of patients prescribed three antibiotics	8	(1.5)
Number (%) of patients who received antimicrobials for empirical therapy	529	(96.4)
Number (%) of antimicrobials prescribed that were consistent with national STGs	1	(0.2)
Number (%) of antimicrobials prescribed that were consistent with facility-based guidelines	502	(94.9)

**Table 5:** Modification in the antimicrobial treatment among neonates admitted with suspected sepsis at Komfo Anokye Teaching Hospital, Ghana, between January 2021 and December 2021.

Modified Treatment	Total *n* (%)
Cefotaxime	46 (67.6)
Ciprofloxacin	13 (19.1)
Ciprofloxacin + metronidazole	3 (4.4)
Cefuroxime + gentamicin	1 (1.5)
Cefuroxime + metronidazole + gentamicin	1 (1.5)
Benzylpenicillin + gentamicin	1 (1.5)
Ampicillin + ciprofloxacin	1 (1.5)
Amikacin	1 (1.5)
Flucloxacillin	1 (1.5)
Total	68 (100)

**Table 6:** Bacterial isolates from blood samples of culture-positive neonates admitted with suspected sepsis at Komfo Anokye Teaching Hospital, Ghana, between January 2021 and December 2021.

Bacterial Isolates	Number	Percentage
**Total**	70	(100)
**Gram-positives**	60	(85.7)
*Coagulase-negative Staphylococcus*	34	(48.6)
*Staphylococcus aureus*	22	(31.4)
*Enterococcus* spp.	3	(4.3)
*Streptococcus* spp.	1	(1.4)
**Gram-negatives**	10	(14.3)
*Coliforms*	4	(5.7)
*Pseudomonas* spp.	3	(4.3)
*Klebsiella* spp.	2	(2.9)
*Escherichia coli*	1	(1.4)

**Table 7:** Antimicrobial resistance patterns of Gram-positive isolates among neonates admitted with suspected sepsis at Komfo Anokye Teaching Hospital, Ghana, between January 2021 and December 2021.

Medicines	Antimicrobial Resistance Pattern									
	*Staphylococcus aureus* (*n* = 22)			*CoNS* (*n* = 34)			*Streptococcus* spp. (*n* = 1)		*Enterococcus* spp. (*n* = 3)	
	N	R	(%) ^†^	N	R	(%) ^†^	N	R	N	R
Penicillin	6	6	(100)	-	-	-	-	-	-	-
Cloxacillin	2	2	(100)	3	3	(100)	-	-	1	1
Gentamicin	18	8	(44.4)	31	13	(41.9)	1	1	3	1
Ciprofloxacin	14	2	(14.3)	32	9	(28.1)	1	1	3	1
Linezolid	2	1	(50)	3	0	(0)	-	-	1	0
Tetracycline	1	1	(100)	-	-	-	-	-	-	-

CoNS = coagulase-negative Staphylococcus; R = resistant; N = total number of isolates; ^†^ row percentages.

**Table 8:** Antimicrobial resistance patterns of Gram-negative isolates from blood samples of neonates admitted with suspected sepsis at Komfo Anokye Teaching Hospital, Ghana, between January 2021 and December 2021.

Medicines	Antimicrobial Resistance Pattern							
	*Klebsiella* spp.* (*n* = 1)		*Escherichia coli* (*n* = 1)		*Coliforms* (*n* = 4)		*Pseudomonas* spp. (*n* = 3)	
	N	R	N	R	N	R	N	R
Ampicillin	1	1	1	1	4	4		
Gentamicin	1	0	1	1	2	2	2	2
Amikacin	1	0	1	0	3	1	2	1
Cefuroxime	1	1	1	1	4	4	-	-
Ceftriaxone	1	1	1	1	3	1	-	-
Cefotaxime	1	1	1	1	4	1	-	-
Ciprofloxacin	1	0	1	0	4	1	2	0
Levofloxacin	1	0	1	0	-	-	-	-
Chloramphenicol	-	-	1	1	4	4	-	-

R = resistant; N = total number of isolates; * only one isolate was tested for resistance against stated medicines.

**Table 9:** Overall status of resistance of isolates to antimicrobials among neonates admitted with suspected sepsis at Komfo Anokye Teaching Hospital, Ghana, between January 2021 and December 2021.

Antimicrobial	Number of Tests	Resistant	
		*n*	%
Gentamicin	59	28	(47.5)
Ciprofloxacin	58	14	(24.1)
Amikacin	56	6	(10.7)
Ampicillin	50	45	(90.0)
Co-trimoxazole	50	33	(66.0)
Cefuroxime	42	27	(64.3)
Erythromycin	42	30	(71.4)
Flucloxacillin	31	30	(96.7)
Cefotaxime	22	19	(86.4)
Levofloxacin	22	4	(18.1)
Ceftriaxone	13	10	(76.9)
Chloramphenicol	13	10	(76.9)
Lincomycin	10	7	(70)
Ofloxacin	9	2	(22.2)
Azithromycin	8	5	(62.5)
Roxithromycin	7	4	(57.1)
Penicillin	6	6	(100)
Cloxacillin	6	6	(100)
Linezolid	6	1	(16.7)
Cephalexin	6	5	(83.3)
Piperacillin + sulbactam	6	5	(83.3)
Amoxicillin	5	5	(100)
Sparfloxacin	5	1	(20.0)
Benzylpenicillin	2	2	(100)
Ampicillin + sulbactam	2	2	(100)
Tetracycline	1	1	(100)

Data were based on number of samples with positive bacterial growth and in which a specific antimicrobial was tested for sensitivity and resistance against antimicrobials.

## Data Availability

The data presented in this study are available on request from the corresponding author. The data are not publicly available due to privacy and ethical concerns.
